# Cost-effectiveness of pazopanib versus sunitinib for metastatic renal cell carcinoma in the United Kingdom

**DOI:** 10.1371/journal.pone.0175920

**Published:** 2017-06-21

**Authors:** Jordan Amdahl, Jose Diaz, Arati Sharma, Jinhee Park, David Chandiwana, Thomas E. Delea

**Affiliations:** 1 Research, Policy Analysis Inc. (PAI), Brookline, Massachusetts, United States of America; 2 Global Health Outcomes − Oncology, GlaxoSmithKline, Stockley Park West, Uxbridge, Middlesex, United Kingdom; 3 Worldwide Health Outcomes, Value & Access, Novartis Pharmaceuticals Corporation, East Hanover, New Jersey, United States of America; 4 Worldwide Health Outcomes, Value & Access, Novartis Pharmaceuticals UK Limited, Camberley, Surrey, United Kingdom; Seoul National University College of Pharmacy, REPUBLIC OF KOREA

## Abstract

**Background:**

Sunitinib and pazopanib are the only two targeted therapies for the first-line treatment of locally advanced or metastatic renal cell carcinoma (mRCC) recommended by the United Kingdom’s National Institute for Health and Care Excellence. Pazopanib demonstrated non-inferior efficacy and a differentiated safety profile versus sunitinib in the phase III COMPARZ trial. The current analysis provides a direct comparison of the cost-effectiveness of pazopanib versus sunitinib from the perspective of the United Kingdom’s National Health Service based on data from COMPARZ and other sources.

**Methods:**

A partitioned-survival analysis model with three health states (alive with no progression, alive with progression, or dead) was used to estimate the incremental cost per quality-adjusted life-year (QALY) gained for pazopanib versus sunitinib over five years (duration of follow-up for final survival analysis in COMPARZ). The proportion of patients in each health state over time was based on Kaplan–Meier distributions for progression-free and overall survival from COMPARZ. Utility values were based on EQ-5D data from the pivotal study of pazopanib versus placebo. Costs were based on medical resource utilisation data from COMPARZ and unit costs from secondary sources. Probabilistic and deterministic sensitivity analyses were conducted to assess uncertainty of model results.

**Results:**

In the base case, pazopanib was estimated to provide more QALYs (0.0565, 95% credible interval [CrI]: −0.0920 to 0.2126) at a lower cost (−£1,061, 95% CrI: −£4,328 to £2,067) versus sunitinib. The probability that pazopanib yields more QALYs than sunitinib was estimated to be 76%. For a threshold value of £30,000 per QALY gained, the probability that pazopanib is cost-effective versus sunitinib was estimated to be 95%. Pazopanib was dominant in most scenarios examined in deterministic sensitivity analyses.

**Conclusions:**

Pazopanib is likely to be a cost-effective treatment option compared with sunitinib as first-line treatment of mRCC in the United Kingdom.

## Introduction

Renal cell carcinomas (RCCs) originate in the renal epithelium and represent approximately 85% of all kidney cancers [1]. The diagnosis of RCC can be further subdivided into clear cell (80–90%), papillary (10–15%), chromophobe (4–5%), and collecting duct carcinoma (1%) [2]. In 2014, RCC was the seventh most common cancer in the United Kingdom (UK), accounting for 12,450 new cases, with 7,800 cases in males and 4,700 cases in females [3]. The overall incidence of RCC in the UK has increased since the 1970s, with the incidence in both sexes increasing with age, particularly after 60 years. According to Cancer Research UK, the five-year survival rate for RCC, including all adults of any age diagnosed with any stage of RCC, is 53.3% for males and 54.8% for females. However, the five-year survival rate for those with metastatic disease is around 10% [2]. Approximately 25–30% of patients with RCC present with symptoms of metastasis (e.g., hemoptysis, bone pain, or pathological fracture) at diagnosis.

Chemotherapy has not been an effective treatment option for RCC, and cytokine therapy with interferon-α lacks effectiveness and causes significant toxicity [4, 5]. Targeted therapies (e.g., sorafenib, bevacizumab, sunitinib, temsirolimus, and everolimus) that act at specific cellular sites have improved progression-free survival (PFS) compared with interferon in both the first- and second-line settings [6]. Consequently, targeted therapy is currently the standard of care in Europe, Canada, the United States (US), and other countries. Sunitinib and pazopanib are the only two targeted therapies recommended for use by the UK’s National Institute for Health and Care Excellence (NICE) for the first-line treatment of advanced and/or metastatic RCC (mRCC) [7, 8].

Pazopanib (Votrient^®^, Novartis, AG, Basel, Switzerland) is a potent multi-targeted tyrosine kinase inhibitor that inhibits all known vascular endothelial growth factor receptors and platelet-derived growth factor receptors [9, 10]. In a placebo-controlled trial (VEG105192; ClinicalTrials.gov identifier: NCT00334282), pazopanib was well tolerated and significantly improved overall PFS versus placebo in the overall population (median PFS: 9.2 vs. 4.2 months; hazard ratio [HR] = 0.46; 95% confidence interval [CI]: 0.34–0.62; *p* < 0.0001) as well as in both treatment-naïve patients (median PFS: 11.1 vs. 2.8 months; HR = 0.40; 95% CI: 0.27–0.60; *p* < 0.0001) and cytokine-pretreated patients (median PFS: 7.4 vs. 4.2 months; HR = 0.54; 95% CI: 0.35–0.84; *p* < 0.001) with advanced RCC and/or mRCC [11]. Pazopanib is approved in the UK as a first-line treatment for adults with advanced RCC, as well as in those who have had prior cytokine therapy for advanced RCC [12].

The COMPARZ trial (Comparing the Efficacy, Safety and Tolerability of Pazopanib versus Sunitinib; NCT00720941) was an open-label phase III study comparing pazopanib versus sunitinib malate (Sutent^®^, Pfizer, Kent, UK) [13] in metastatic clear-cell RCC; COMPARZ was the largest head-to-head study conducted in this setting to date. The trial was conducted in 14 countries in Europe (Germany, Ireland, Italy, the Netherlands, Spain, Sweden, and the UK), Asia (China, Japan, Korea, and Taiwan), North America (Canada and the US), and Australia. Eligibility criteria included adult patients with advanced RCC or mRCC with a clear-cell histologic component who had not received previous systemic treatment. Patients were excluded if they had a Karnofsky Performance Status score of < 70; inadequate organ system function; a history of another malignancy or central nervous system metastases; poorly controlled hypertension; a history of cardiovascular disease, cerebrovascular accident, or pulmonary embolism within 12 months of enrollment; known endobronchial lesions and/or lesions infiltrating major pulmonary vessels; or any other serious pre-existing condition that could interfere with the patient’s safety or provision of informed consent.

A total of 1,100 patients were randomised to receive either four-week cycles of pazopanib 800 mg once daily (n = 557) or six-week cycles of sunitinib 50 mg once daily for four weeks followed by two weeks without treatment (n = 553). The study was powered to demonstrate the non-inferiority of pazopanib versus sunitinib with respect to independent review committee-assessed PFS based on a predefined non-inferiority margin that was defined to exclude a difference in the HRs greater than 25% (i.e., upper bound of the 95% CI < 1.25). Overall survival (OS), safety, quality of life, and medical resource utilisation (MRU) were secondary endpoints. Based on analyses of data through a clinical data cut-off date of 21 May 2012, pazopanib was non-inferior to sunitinib with respect to PFS (HR = 1.05; 95% CI: 0.90–1.22). The OS rate was also similar for the two groups (HR = 0.91; 95% CI: 0.76–1.08).

Patients receiving pazopanib had a lower incidence of fatigue (55% vs. 63%; relative risk ratio [RRR] = 0.87; 95% CI: 0.79–0.96), hand–foot syndrome (30% vs. 50%; RRR = 0.59; 95% CI: 0.50–0.68), and thrombocytopenia (10% vs. 34%; RRR = 0.30; 95% CI: 0.23–0.40) compared with those receiving sunitinib, although they had a higher incidence of elevated alanine aminotransferase (31% vs. 18%; RRR = 1.74; 95% CI: 1.40–2.17) compared with those receiving sunitinib [13, 14]. The mean change from baseline in 11 of 14 health-related quality-of-life (HRQOL) domains favored pazopanib over sunitinib [13]. Three of these differences between the two agents had effect sizes of less than 0.20, while seven differences had effect sizes between 0.20 and 0.50 (viewed as small-to-medium by convention); the effect size for the eleventh difference—mouth and throat soreness—fell within the medium-to-large range (conventionally 0.50 to 0.80).

The MRU data were prospectively collected in the COMPARZ study beginning at day 28 of every cycle through cycle 9. During cycle 10 and beyond, data were collected at day 42 of every cycle until treatment discontinuation. Data collected included medical office visits, laboratory visits and tests, home healthcare, hospitalisation, urgent care, and medical/surgical procedures. In a protocol-specified analysis of MRU out to 24 weeks on study, patients in the pazopanib arm had nominally lower mean monthly utilisation of non-study medical visits and hospital days, as well as significantly lower mean monthly utilisation of telephone consultations and emergency department (ED) visits compared with patients in the sunitinib arm [13].

The PISCES trial (Patient Preference Study of Pazopanib versus Sunitinib in Advanced or Metastatic Kidney Cancer; NCT01064310) was a randomised, double-blind, multicentre, crossover phase IIIb study that evaluated patients’ preferences between pazopanib and sunitinib and the impact of HRQOL and safety factors on these preferences. In PISCES, treatment-naïve patients with locally advanced RCC or mRCC and their physicians were asked to assume that both treatments were equally effective and to state which drug they preferred to continue treatment with, or whether they preferred neither drug. The majority of both patients (70% vs. 22%) and physicians (61% vs. 22%) preferred pazopanib over sunitinib, primarily because it offered better quality of life and less fatigue [15, 16].

In the European Union, pazopanib was originally issued with a conditional license, pending results from the COMPARZ trial and demonstration of non-inferiority to sunitinib [7]. A conditional marketing authorisation is granted to a medicinal product for which the balance between risks and benefits is assessed to be positive based on the available evidence. Additionally, the product needs to fulfill an unmet medical need, and the benefit to the public health of its immediate availability has to outweigh any potential risks due to the lack of available data at that time. In order to achieve reimbursement in England and Wales through the UK’s National Healthcare System (NHS) (as recommended by NICE) and in Scotland through the Scottish Medicines Consortium, an innovative patient access scheme (PAS) was developed [7]. The PAS had two goals: (1) to ensure that the medication costs of pazopanib were equivalent to those of sunitinib, and (2) to provide a risk-sharing arrangement between the manufacturer and the NHS such that, in the event of pazopanib’s non-inferiority not being demonstrated by the COMPARZ trial, there would be a partial rebate of medication costs to the NHS. This two-part PAS allowed patients early access to and reimbursement for pazopanib. The success of the COMPARZ study in demonstrating the non-inferiority of pazopanib has subsequently resulted in full marketing authorisation being granted by the European Medicines Agency and—following the agreement of NICE, the Scottish Medicines Consortium, and the Department of Health—the removal of the partial rebate of medication costs provision (part 2) in the initial PAS; however, the 12.5% discount (part 1) remains in place.

Together, the COMPARZ and PISCES trials provide evidence of the non-inferior efficacy, differentiated safety profile, enhanced quality of life, lower MRU costs, and greater patient and physician preference for pazopanib over sunitinib. However, a cost-effectiveness analysis of pazopanib versus sunitinib was not included as part of these studies. Two previous studies have evaluated the cost-effectiveness of pazopanib versus sunitinib in the first-line treatment of patients with mRCC based on COMPARZ from the US [17] and Canadian [18] healthcare system perspectives. In the current study, the cost-effectiveness of pazopanib versus sunitinib was evaluated from a UK healthcare system perspective.

## Materials and methods

### Overview

Cost-effectiveness was evaluated using a partitioned-survival model that defined health states based on disease progression and survival. Patients were assumed to be in one of three mutually exclusive health states: pre-progression, post-progression, or dead. The population of interest was treatment-naïve patients with mRCC consistent with that of the COMPARZ trial [13]. The median age of patients in COMPARZ was 61 and 62 years in the pazopanib and sunitinib groups, respectively, and > 70% of patients were male. Prior nephrectomy had been performed in about 83% of patients, and most patients were at intermediate risk (Memorial Sloan Kettering Cancer Center risk criteria; 58% in the pazopanib group vs. 59% in the sunitinib group). The results of subgroup analyses in COMPARZ provided no evidence of the heterogeneity of treatment effects [13].

A UK healthcare system perspective was employed. Only costs related to the treatment of mRCC that were likely to be directly affected by treatment with either of the comparators were considered. Only pazopanib and sunitinib were included as comparators, as these are the only two targeted therapies recommended for use by NICE for the first-line treatment of mRCC [7, 8]. The model time horizon for the base case was set to five years based on the maximum follow-up time for which data were available for OS rates in the COMPARZ trial. In sensitivity analyses, a 10-year time horizon was used to approximate a lifetime projection (at 10 years, > 95% of all patients are projected to be dead). Costs and HRQOL were assumed to be conditioned on treatment and expected time in these disease states. The treatment cycle length used in the model was one week in order to accommodate the four-week cycle for pazopanib and the six-week cycle for sunitinib. This relatively short cycle length avoids the need for half-cycle corrections.

The model was used to generate expected lifetime costs for sunitinib and pazopanib. Costs were adjusted to 2014 values. Pre-progression, post-progression, overall life expectancy (i.e., progression-free, post-progression, and overall life-years), and quality-adjusted life-years (QALYs) were also estimated for each comparator. Both costs and QALYs were discounted at an annual rate of 3.5% [19]. The incremental cost-effectiveness ratio was defined as the ratio of the difference in total costs to the difference in QALYs for sunitinib versus pazopanib. The net monetary benefit (NMB) [20] of pazopanib versus sunitinib was also evaluated at cost-effectiveness threshold values of £20,000, £30,000, and £50,000 per QALY.

No institutional review board approval was performed, as this study was a cost-effectiveness analysis of previously approved clinical trials using publicly available information. The model used in this evaluation was also used in an evaluation of the cost-effectiveness of pazopanib versus sunitinib in treatment-naïve mRCC patients in the US [17] and Canada [18]. Different utility values were used in the US analysis [17] than in the Canadian [18] and current analyses. The unit costs estimates used in this analysis differ from those used in both the US and Canadian analyses. Additional details regarding the model can be found in those reports [17, 18].

### Model estimation

#### Progression-free and overall survival

Survival functions for PFS and OS were estimated using data from the COMPARZ trial. In the base-case analysis, Kaplan–Meier distributions were used up to the maximum follow-up time in COMPARZ. Because PFS was not updated in the final analysis of OS in COMPARZ, Kaplan–Meier estimates of PFS were available only for 36 months. It was therefore necessary to project PFS from 36 to 60 months in the base-case analysis. Also, sensitivity analyses using a 10-year time horizon required projections of both PFS and OS beyond five years. Projections of PFS and OS beyond the end of the follow-up period were obtained by fitting a variety of parametric survival distributions (i.e., exponential, Weibull, log-logistic, log-normal, and gamma) to individual patient data from COMPARZ using accelerated failure time regression. Distributions were evaluated based on fit statistics (i.e., Akaike information criterion) and visual inspection. The one-parameter exponential model provided the worst fit, whereas the three-parameter gamma distribution provided the best fit. However, the gamma distribution was not used because the long tails of the gamma distribution were deemed implausible. The two-parameter models (Weibull, log-normal, and log-logistic) all fit the PFS and OS curves similarly. The Weibull model was used for both treatment arms and for both PFS and OS because it provided a good fit to both PFS and OS. The parameters used in the model are published elsewhere [17]. Investigator-assessed PFS was used because this was considered more likely to reflect the assessment of progression in typical clinical practice, and because independent review committee-assessed PFS may be biased by informative censoring for patients with investigator-assessed progression before independent review committee-assessed PFS [21]. A sensitivity analysis was conducted using independent review committee-assessed PFS.

#### Utility values

A preference-based measure of HRQOL was not included in the COMPARZ trial. Mean utility values for pazopanib and sunitinib during PFS were therefore estimated by combining data on the incidence and duration of adverse events (AEs) from COMPARZ with a regression equation relating the presence of AEs to utility values [18]. The regression equation was estimated using generalised linear model (GLM) regression and data from the placebo-controlled VEG105192 trial [11]. The regression equation had EQ-5D utility values as the dependent variable and baseline patient characteristics (age [< 65 or ≥ 65 years], sex, performance status, and prior treatment [yes or no]), treatment group, and the presence of AEs as independent variables. The AEs were characterized by grade (Grade 1–2 vs. ≥ 3) and whether the AE was observed more frequently in the sunitinib arm of COMPARZ. Tests of interaction between AEs and treatment group (pazopanib vs. placebo) were not significant, so data for both pazopanib and placebo patients in VEG105192 were used. This estimated regression equation from VEG105192 and patient-level data on baseline patient characteristics and the incidence and duration of AEs from COMPARZ were then used to estimate utility values for every day of pre-progression follow-up for all patients in COMPARZ. Mean utility values for PFS were then estimated for each treatment group using Kaplan–Meier Sample Average (KMSA) methods. Standard errors (SEs) for utility values were obtained by bootstrapping. Patients were not followed consistently after progression in the VEG10592 trial. Therefore, it was not possible to estimate utility values for the post-progression state using data from this study. The mean utility value for the post-progression state was therefore assumed to be the same for pazopanib and sunitinib, and this was estimated to be 0.5509 based on the utility value for best supportive care after termination of second-line therapy based on data from the sunitinib 014 phase III trial, as reported in an economic evaluation of sunitinib for first-line treatment of mRCC from a Canadian public healthcare system perspective [22].

#### Costs

Costs considered in the model were based on 2014 prices and included the costs of treatment initiation, medication, and dispensing for pazopanib and sunitinib, pre-progression follow-up and monitoring, other mRCC-related care associated with pazopanib and sunitinib treatment during PFS, post-progression supportive care, and in a sensitivity analysis, post-treatment anti-cancer therapy (PTACT). Cost estimates used in the model are presented in Table 1 and described below.

**Table 1:** Cost estimates used in the model.

Service	Mean Cost (£)	
	Pazopanib	Sunitinib
Treatment initiation, per patient	48.69	48.69
Medication, per day	74.72	112.10
Dispensing, per prescription	14.74	14.74
Pre-progression follow-up and monitoring, per month	153.48	153.48
Post-progression supportive care, per month	225.08	225.08
Other mRCC-related care associated with pazopanib and sunitinib treatment, per month, pre-progression		
Hospital days	79.16	112.28
Medical office visits	12.07	12.76
Medical/surgical specialty visits	33.74	38.00
Telephone consultations	1.68	1.57
Emergency department visits	2.75	4.38
Home healthcare visits	0.77	3.14
Laboratory visits	1.87	2.41
Laboratory tests	4.78	4.45
Radiology tests	17.32	20.78
Total	154.14	199.77
PTACT, per patient with progression		
Axitinib	895.53	1,296.64
Bevacizumab	1,026.32	829.79
Everolimus	3,374.76	3,259.21
Pazopanib	364.23	880.48
Sirolimus	3.85	3.85
Sorafenib	1,422.97	2,340.99
Sunitinib	2,690.55	1,487.20
Temsirolimus	592.01	844.75
Any cytokine (assumed to be IFN-α)	122.99	95.91
Other (assigned same cost as IFN-α)	226.14	183.82
Unlicensed	0	0
Total	10,899.37	11,222.67

IFN, interferon; mRCC, metastatic renal cell carcinoma; PTACT, post-treatment anti-cancer therapy.

Planned dosages of pazopanib and sunitinib were assumed to be similar to those used in the COMPARZ trial. Unit costs of pazopanib and sunitinib were provided by Novartis. The list price of pazopanib was adjusted to reflect the 12.5% discount from the list price as part of the PAS [7]. Although no discount to the list price of sunitinib was assumed, Pfizer (the manufacturer of sunitinib) has agreed to a PAS with the NHS in which the first treatment cycle of sunitinib (i.e., 28 days of treatment in the first six weeks) is provided at no cost to the NHS [8]. Based on the planned dosage and the list prices (but not the PAS for pazopanib or sunitinib), the cost per six weeks of therapy was the same for pazopanib and sunitinib (£3,139). The average daily costs of pazopanib and sunitinib over six weeks were £74.72. With the 12.5% discount offered to the NHS as part of the PAS for pazopanib, the average cost per six weeks of therapy with pazopanib was £2,745.96, and the average daily cost was £65.38. The sunitinib discount was incorporated into the model as a negative one-off cost of £3,139 at the time of therapy initiation.

Pazopanib was assumed to be prescribed every four weeks, whereas sunitinib was assumed to be prescribed every six weeks. Patients receiving pazopanib were assumed to incur the cost of a 28-day supply each 28-day cycle while they remained alive and progression-free, while those receiving sunitinib were assumed to incur the cost of a 28-day supply each 42-day cycle while they remained alive and progression-free. Any medication supplied but not taken was assumed to be discarded. The model assumed that there were no costs associated with administering pazopanib and sunitinib, although each prescription was assumed to have a dispensing cost of £14.74 based on the cost of 15 minutes of time for a community pharmacist (hourly rate of a community pharmacist: £58.96 ÷ 4 = £14.74) [23].

By default, the model calculates the amount of medication and number of prescriptions based on the planned dosage of the medication and the distribution of PFS. However, the model also includes two adjustment factors that can be used to scale medication and dispensing costs up or down, since the actual amount of medication and number of dispensations may differ owing to dose reductions, treatment interruptions, and differences between the distribution of time to discontinuation and the distribution of PFS. The adjustment factors for medication costs for pazopanib and sunitinib (68.5% and 67.0%, respectively) were estimated based on the ratio of the mean actual versus planned cumulative dose with pazopanib and sunitinib in COMPARZ. Mean actual dose was calculated as the sum, over all days of follow-up, of the product of Kaplan–Meier estimates of time to discontinuation on each day of follow-up and the mean dose received among all patients remaining uncensored and on therapy for each day of follow-up. Mean planned dose was calculated as the sum, over all days of follow-up, of the product of Kaplan–Meier estimates of PFS on each day of follow-up and the mean dose that patients would have received on that day had all patients received the planned dose of the medication. The adjustment factors for dispensing costs for pazopanib and sunitinib (78.1% and 79.4%, respectively) were estimated using the ratio of the mean actual versus planned number of days of pazopanib and sunitinib therapy in COMPARZ. Mean actual days of therapy were calculated as the sum, over all days of follow-up, of the product of Kaplan–Meier estimates of time to discontinuation on each day of follow-up and an indicator function that equals 1 on days when the patient received pazopanib and 0 otherwise. Mean planned number of doses was calculated as the sum, over all days of follow-up, of the product of Kaplan–Meier estimates of PFS on each day of follow-up and an indicator function that equals 1 on days when the patient was scheduled (per protocol) to receive treatment and 0 otherwise.

The costs of pre-progression routine care and follow-up and post-progression supportive care, were estimated based on the costs reported in the NICE Evidence Review Group evaluation of pazopanib as first-line treatment for patients with mRCC conducted for the NICE Technology Appraisal Guidance 215 [7]. The monthly cost of pre-progression follow-up and monitoring was calculated as the sum of the cost of a consultant-led outpatient visit (one visit per month) and one third of the cost of a computed tomography (CT) scan (one test every three months). The monthly cost of post-progression supportive care was estimated as the sum of the costs of a visit to a general practitioner, a consultation with a community nurse, and one month's supply of morphine sulphate. The cost of treatment initiation was calculated as the difference between the costs of the initial and the subsequent consultant-led visits. The SEs of the cost estimates were assumed to be 25% of the mean estimates.

Costs of other mRCC-related care associated with pazopanib and sunitinib treatment were estimated on a monthly basis by combining the estimates of monthly non-study MRU from the COMPARZ trial with the unit cost estimates from published or publicly available sources. These costs were assumed to be incurred each month prior to progression.

In a *post-hoc* analysis of data from the COMPARZ trial, overall monthly MRU was assessed for the entire study period [24]. Each patient’s MRU was summed over their individual follow-up period and then divided by the mean follow-up (in months) for the intention-to-treat population (average follow-up: 10.4 months for pazopanib vs. 10.8 months for sunitinib) to obtain monthly estimates. For most measures of MRU, mean monthly utilisation was greater with sunitinib versus pazopanib, although these differences were statistically significant only for radiological visits (mean 0.072 vs. 0.050 per month; *p* = 0.04) and ED visits (mean 0.035 vs. 0.022 per month; *p* = 0.003) [24].

The model took into consideration the costs of non-study medical office visits, laboratory visits and tests, radiological visits and tests, home healthcare visits, outpatient visits, and hospital days (general ward or intensive care unit [ICU] days). Costs of general ward and ICU days were based on the national average costs of these services from the National Schedule of Reference Costs for 2011–2012 for NHS trusts and NHS foundation trusts [25], adjusted to 2014 prices using the Consumer Price Index (CPI) for health [26]. The cost per day in the general ward was calculated as a weighted average of the average daily costs of non-elective inpatient long stays and excess bed days for Healthcare Resource Group (HRG) codes LB06D (kidney, urinary tract or prostate neoplasms, with length of stay two days or more, with major complications), LB06E (kidney, urinary tract or prostate neoplasms, with length of stay two days or more, with intermediate complications), and LB06G (kidney, urinary tract or prostate neoplasms, with length of stay one day or less), with HRGs weighted by number of days. The unit cost of ICU days was calculated as a weighted average of the average daily costs of adult critical care services for HRG codes XC01Z–XC07Z (adult critical care, 0–6 or more organs supported), with HRGs weighted by the number of bed days.

The costs of ED visits were based on NHS Reference Costs for outpatient attendances for HRG 180: Accident & Emergency. The costs of laboratory visits and laboratory and radiology tests were based on the NHS Reference Costs for Direct Access Pathology Services [26], or Unit Costs of Health and Social Care 2012 estimates from the Personal Social Services Research Unit, if available [23]. The cost of a urinalysis test was as published by the NHS Centre for Evidence-based Purchasing [27]. The costs of a positron-emission tomography (PET), PET/CT scans, and tumor marker testing were as reported by Auguste et al. [28]. Estimates from prior years were adjusted to 2014 prices using the CPI for health [26]. The cost of a laboratory visit was assumed to be the same as the cost of a nurse visit. The costs of “other” services or tests were assigned the average cost of related specific services or tests. The mean monthly other costs associated with pazopanib and sunitinib treatment were estimated to be £154 and £199, respectively, suggesting a nominal difference in cost that is £45 per month higher for sunitinib versus pazopanib.

The medication costs of PTACT were estimated using data on the utilisation of PTACT in the COMPARZ trial and published costs estimates. Medication costs for PTACTs were based on prices from the British National Formulary. The PAS for sunitinib or pazopanib was assumed to apply when either agent was used as a second-line therapy. The estimated costs of PTACT were similar for pazopanib and sunitinib (£10,899 and £11,223, respectively, per patient with progression).

### Analyses

Probabilistic sensitivity analyses involved simultaneous sampling from estimated probability distributions of model parameters to obtain 1,000 sets of model input estimates [29, 30]. Both PFS and OS were sampled from bootstrapped Kaplan–Meier survival distributions. In cases of sampled distributions of PFS and OS in which the maximum follow-up time was less than 37.5 and 60.30 months, respectively, PFS and OS were estimated from the end of follow-up extrapolation using Weibull distributions fitted to the data from the corresponding bootstrap sample. The utility for PFS was assumed to be distributed as a beta random variable, and the decrement in utility for post-progression survival versus PFS was assumed to be distributed as a normal random variable. Unit costs of pazopanib and sunitinib, dispensing, and administration were not sampled. Costs of treatment initiation, pre-progression follow-up and monitoring, and post-progression supportive care were assumed to be distributed as log-normal random variables with SEs assumed to be 0.25 times the base-case estimate. Non-study MRU costs from the COMPARZ study [24] were distributed as log-normal variables with SEs for these costs derived from those for MRU estimates. The expected costs and QALYs for each simulation were calculated for pazopanib and sunitinib, along with the differences between pazopanib and sunitinib in expected costs and QALYs and the incremental cost per QALY gained with pazopanib versus sunitinib. Ninety-five percent credible intervals (95% CrI) [31] were calculated for expected costs and QALYs and the differences in expected costs and QALYs based on the 2.5th and 97.5th percentiles of these simulations. For each comparison, simulation results were plotted on the cost-effectiveness plane, and cost-effectiveness acceptability curves were constructed for pazopanib versus sunitinib to identify the proportion of simulations in which pazopanib was preferred given various levels of decision-makers' threshold values for cost per QALY gained [32].

The impact of changing assumptions concerning key model parameter values on the incremental cost-effectiveness and NMB of pazopanib versus sunitinib was explored through scenario analyses, which incorporated a variety of different scenarios encompassing variations in the time horizon, PFS, OS, relative dose intensity, administration/dispensing costs, monthly costs, decrements in utility, and discount rates. Several scenarios involved setting the effectiveness, estimates, costs, or utility values for sunitinib equal to those for pazopanib to assess the impact of the assumed differences between treatments in these parameters. In the absence of alternative estimates from similar studies, costs, dose-intensity estimates, and disutilities were varied by ± 50% of base-case values. When calculating NMB, the threshold value of cost-effectiveness was defined as £30,000 per QALY, with a positive value for NMB suggesting that pazopanib is cost-effective at the threshold value.

## Results

In the base case, pazopanib was estimated to yield 0.0135 fewer progression-free life-years (discounted) than sunitinib (1.1533 and 1.1668, respectively) and 0.0708 more post-progression life-years (discounted) than sunitinib (1.14251 and 1.3542, respectively) (Table 2). Discounted life-years were estimated to be 0.0574 higher with pazopanib compared with sunitinib (2.5784 and 2.5210, respectively). Discounted QALYs were estimated to be 0.0595 greater with pazopanib versus sunitinib (95% CrI: −0.0920 to 0.2126). Discounted pre-progression QALYs were 0.0204 higher with pazopanib, and discounted post-progression QALYs were 0.0390 higher with pazopanib.

**Table 2:** Base-case results*.

Outcomes	Pazopanib	Sunitinib	Pazopanib vs. Sunitinib*
**Effectiveness, discounted**			
Life-years			
Pre-progression	1.1533	1.1668	−0.0135
Post-progression	1.4251	1.3542	0.0708
Total	2.5784	2.5210	0.0574
QALYs			
Pre-progression	0.8176	0.7971	0.0204
Post-progression	0.7851	0.7461	0.0390
Total	1.6026	1.5432	0.0595
**Costs, discounted, £**			
Pazopanib and sunitinib medication 1	19,220	22,528	–3,308
Pazopanib and sunitinib medication 2	0	–3,139	3,139
Total medication costs	19,220	19,389	–170
Pazopanib and sunitinib dispensing	177	125	52
Other pre-progression costs	4,306	4,994	–689
Other post-progression costs	14,423	14,529	–106
Total	38,126	39,038	–912
**Cost-effectiveness, £**			
Cost/PFLYs			67,808^†^
Cost/life-years			Dominant
Cost/QALYs			Dominant
**NMB by cost-effectiveness threshold**			
£20,000 per QALY gained			2,102
£30,000 per QALY gained			2,696
£50,000 per QALY gained			3,886

NMB, net monetary benefit; PFLYs, progression-free life-years; QALYs, quality-adjusted life-years.

*Values were summed before rounding.

^†^Sunitinib was more costly and more effective than pazopanib.

Medication costs were estimated to be £170 greater with sunitinib than with pazopanib (£19,389 vs. £19,220, respectively) (Table 2). This savings reflects in part the lower expected progression-free life-years with pazopanib versus sunitinib (which results in a shorter projected duration of therapy). Dispensing costs were estimated to be £52 higher with pazopanib, owing mostly to its assumed shorter cycle duration (four weeks vs. six weeks for sunitinib). Other costs during PFS were estimated to be £689 lower with pazopanib, largely reflecting the lower monthly costs associated with pazopanib versus sunitinib treatment based on MRU data from the COMPARZ trial. Other costs during post-progression survival (including costs of PTACT) were estimated to be £106 lower with pazopanib. Expected total costs were therefore £912 lower with pazopanib versus sunitinib (95% CrI: −£4,328, £2,067). Because pazopanib was estimated to provide more QALYs at a lower cost compared with sunitinib, it was found to be dominant compared with sunitinib in the base-case analysis.

At threshold values of cost-effectiveness of £20,000, £30,000, and £50,000 per QALY gained, the NMB values for pazopanib versus sunitinib were £2,102 (95% CrI: −£220 to £4,673), £2,696 (95% CrI: −£677 to £6,167), and £3,886 (95% CrI: −£1,829 to £10,037), respectively. At a threshold value of cost-effectiveness of £30,000 per QALY gained, 66% of the NMB of £2,696 was a consequence of increased QALYs (0.0595 QALYs gained “monetized” at a value of £30,000 per QALY equals £1,784) and 34% of the NMB was a consequence of reduced costs (savings of £912).

**Fig 1:**
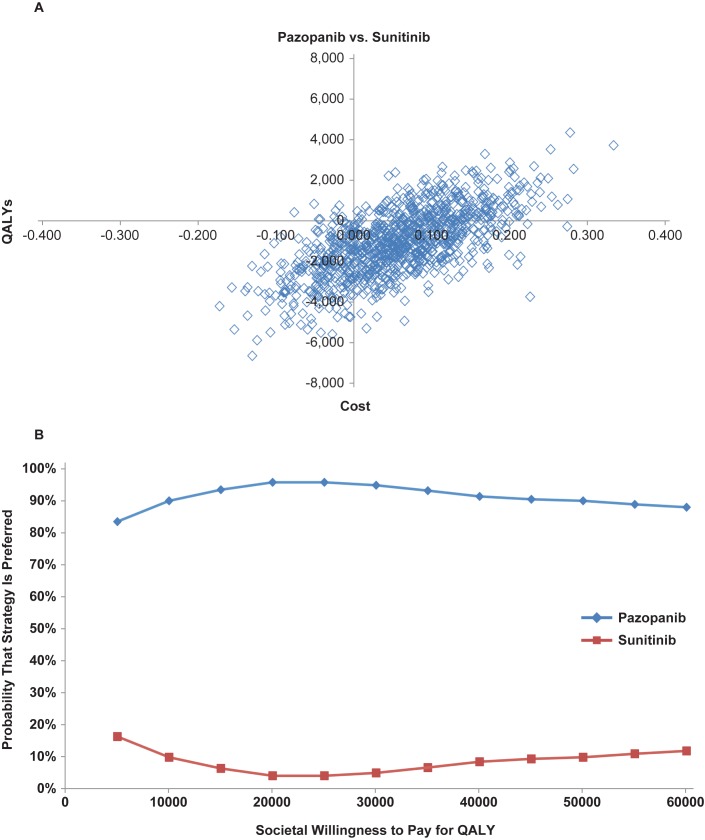
**Results of probabilistic sensitivity analyses; (A) cost-effectiveness plane and the (B) cost-effectiveness acceptability curves.** QALY, quality-adjusted life-year.

Based on probabilistic sensitivity analyses, the probability that pazopanib would yield more QALYs than sunitinib was estimated to be 76%, while the probability that pazopanib would have lower expected costs than sunitinib was estimated to be 74% (Fig 1A). In 51% of all simulations, pazopanib was projected to yield more QALYs and lower costs compared with sunitinib (pazopanib dominant). In 1% of all simulations, pazopanib was projected to yield higher costs and lower QALYs (sunitinib dominant). The probability that pazopanib is cost-effective versus sunitinib was estimated to be 96% and 95% for the threshold values of cost-effectiveness of £20,000 and £30,000 per QALY gained, respectively (Fig 1B).

**Fig 2:**
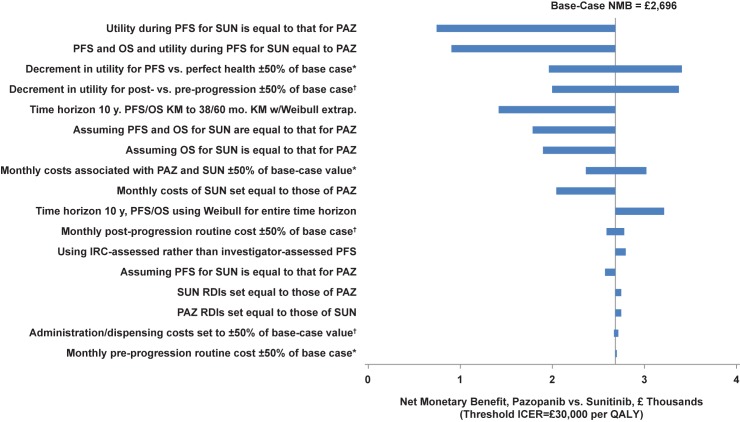
Tornado diagram for the NMB of pazopanib versus sunitinib. IRC, independent review committee; KM, Kaplan–Meier; NMB, net monetary benefit; OS, overall survival; PAZ, pazopanib; PFS, progression-free survival; PTACT, post-treatment anti-cancer therapy; QALY, quality-adjusted life-year; RDIs, relative dose intensities; SUN, sunitinib. *Low value of parameter corresponds to low value of NMB; high value of parameter corresponds to high value of NMB. ^†^Low value of parameter corresponds to high value of NMB; high value of parameter corresponds to low value of NMB.

Pazopanib was dominant in almost all scenarios examined in deterministic sensitivity analyses (Table 3). The probability that pazopanib was cost-effective versus sunitinib at a threshold incremental cost-effectiveness ratio of £30,000 was not less than 50% in any scenario. The tornado diagram for NMB (with the threshold of cost-effectiveness set at £30,000) for pazopanib versus sunitinib is shown in Fig 2. The NMB for pazopanib compared with sunitinib ranged from £752 (assuming utility values during PFS for sunitinib were equal to those of pazopanib) to £3,417 (decrement in utility for PFS vs. perfect health set to 150% of the base case). The NMB was most sensitive to assumptions regarding the utility values and the distributions of PFS and OS. The NMB of pazopanib versus sunitinib was higher when the time horizon of 10 years was used and PFS and OS were modelled using a Weibull distribution for the entire time horizon. The NMB of pazopanib versus sunitinib was relatively insensitive to changes in the other parameters and assumptions. In no scenario was the NMB of pazopanib < £0.

**Table 3:** Summary of results from scenario analyses.

Scenario		Deterministic Results						Probabilistic Results				
		Difference, PAZ vs. SUN		ICER (£)	NMB, by WTP for QALY (£)			Probability that Therapy is Dominant		Probability that Pazopanib is Cost-Effective, by WTP for QALY		
No.	Description	Costs (£)	QALYs		20,000	30,000	50,000	PAZ	SUN	£20,000	£30,000	£50,000
1	Base Case	−912	0.06	Dominant	2,101	2,696	3,886	51%	1%	96%	95%	90%
2	Time horizon set to 10 years. PFS and OS modelled using Kaplan–Meier to 5 years with Weibull extrapolation thereafter	−1,244	0.01	Dominant	1,366	1,428	1,550	28%	4%	76%	67%	59%
3	Time horizon set to 10 years. PFS and OS modelled using Weibull distribution for entire model time horizon	−1,164	0.07	Dominant	2,535	3,220	4,591	97%	0%	100%	100%	99%
4	Assuming PFS for sunitinib is equal to that for pazopanib	−747	0.06	Dominant	1,972	2,585	3,809	49%	2%	89%	90%	88%
5	Assuming OS for sunitinib is equal to that for pazopanib	−1,067	0.03	Dominant	1,624	1,903	2,460	46%	2%	87%	84%	78%
6	Assuming PFS and OS for sunitinib are equal to that for pazopanib	−902	0.03	Dominant	1,495	1,791	2,384	35%	4%	83%	81%	76%
7	Using IRC-assessed rather than investigator-assessed PFS	−875	0.06	Dominant	2,165	2,810	4,100	46%	1%	92%	92%	91%
8	Pazopanib RDIs set equal to those of sunitinib	−964	0.06	Dominant	2,154	2,748	3,938	54%	1%	95%	95%	91%
9	Sunitinib RDIs set equal to those of pazopanib	−976	0.06	Dominant	2,165	2,760	3,949	54%	0%	96%	97%	94%
10	Administration/ dispensing costs set to 50% of base-case value	−938	0.06	Dominant	2,128	2,722	3,912	55%	1%	94%	95%	91%
11	Administration/ dispensing costs set to 150% of base-case value	−886	0.06	Dominant	2,075	2,670	3,859	53%	0%	97%	96%	92%
12	Monthly costs associated with pazopanib and sunitinib set to 50% of base-case value	−580	0.06	Dominant	1,770	2,364	3,554	51%	1%	95%	94%	90%
13	Monthly costs associated with pazopanib and sunitinib set to 150% of base-case value	−1,244	0.06	Dominant	2,433	3,028	4,217	51%	1%	95%	94%	92%
14	Monthly costs associated with sunitinib set to those for pazopanib	-273	0.06	Dominant	1,463	2,057	3,247	54%	1%	96%	95%	91%
15	Monthly pre-progression routine cost set to 50% of base-case value	−900	0.06	Dominant	2,089	2,684	3,873	52%	0%	95%	95%	91%
16	Monthly pre-progression routine cost set to 150% of base-case value	−925	0.06	Dominant	2,114	2,709	3,898	52%	0%	96%	95%	91%
17	Monthly post-progression routine cost set to 50% of base-case value	−1,008	0.06	Dominant	2,197	2,792	3,981	53%	1%	96%	95%	91%
18	Monthly post-progression routine cost set to 150% of base-case value	−816	0.06	Dominant	2,006	2,601	3,790	52%	1%	95%	95%	91%
19	Decrement in utility for PFS vs. perfect health set to 50% of base-case value	−912	0.04	Dominant	1,621	1,975	2,683	51%	1%	95%	94%	91%
20	Decrement in utility for PFS vs. perfect health set to 150% of base-case value	−912	0.08	Dominant	2,582	3,417	5,088	42%	1%	94%	90%	83%
21	Decrement in utility for post- vs. pre-progression set to 50% of base-case value	−912	0.08	Dominant	2,561	3,386	5,035	61%	0%	97%	97%	96%
22	Decrement in utility for post- vs. pre-progression set to 150% of base-case value	−912	0.04	Dominant	1,642	2,006	2,736	54%	1%	96%	95%	91%
23	Assuming utility during PFS for sunitinib is equal to that for pazopanib	−912	−0.01	171,379	806	752	646	47%	1%	96%	96%	91%
24	Assuming PFS and OS and utility during PFS for sunitinib are equal to that for pazopanib	−902	0.00	Dominant	902	902	902	38%	2%	89%	86%	78%
25	Time horizon set to 10 years. PFS and OS modelled using Weibull distribution for entire model time horizon, utility during PFS for sunitinib are equal to that for pazopanib	−1,164	−0.01	170,156	1,027	959	822	20%	6%	69%	64%	58%
26	Time horizon set to 10 years. PFS and OS modelled using Weibull distribution for entire model time horizon, PFS and OS and utility during PFS for sunitinib are equal to that for pazopanib	−708	−0.05	14,664	−258	−740	−1,706	88%	0%	100%	98%	96%
27	Discount rate set to 0%	−1,020	0.06	Dominant	2,239	2,849	4,069	52%	0%	85%	77%	68%
28	Discount rate set to 6%	−841	0.06	Dominant	2,010	2,595	3,764	53%	1%	96%	95%	91%

ICER, incremental cost-effectiveness ratio; IRC, independent review committee; NMB, net monetary benefit; OS, overall survival; PAZ, pazopanib; PFS, progression-free survival; QALY, quality-adjusted life-year; RDI, relative dose intensity; SUN, sunitinib; WTP, willingness to pay.

## Discussion

In COMPARZ, pazopanib showed non-inferior efficacy but a different safety profile versus sunitinib in the first-line treatment of mRCC [13, 16]. Pazopanib and sunitinib were essentially similar with regard to both PFS and OS [13]. The median PFS was 10.5 months (95% CI: 8.3–11.1) with pazopanib and 10.2 months (95% CI: 8.3–11.1) with sunitinib (HR = 1.00; 95% CI: 0.86–1.15). The HR for OS with pazopanib versus sunitinib was 0.91 (95% CI: 0.76–1.08; *p* = 0.28 using a stratified log-rank test). In PISCES, the only other controlled trial comparing pazopanib versus sunitinib as a first-line treatment of mRCC, a majority of patients (70% vs. 22%) and physicians (61% vs. 22%) preferred pazopanib over sunitinib, primarily because it offered better quality of life and less fatigue [15, 16].

The current study evaluated the cost-effectiveness of pazopanib versus sunitinib as the first-line treatment for mRCC from a UK healthcare system perspective using PFS, OS, and MRU data from COMPARZ. Utility values were estimated using data on AEs from COMPARZ and a regression equation based on EQ-5D assessments from the pivotal trial of pazopanib versus placebo in mRCC. In the base-case analyses, pazopanib was projected to yield more QALYs and lower treatment costs compared with sunitinib. Pazopanib was therefore found to be dominant compared with sunitinib in the base case. The benefits of pazopanib on QALYs were derived principally from the assumed difference in HRQOL among patients receiving pazopanib compared with sunitinib, as well as a projected gain in post-progression survival. For a threshold value of £30,000 per QALY gained, the probability that pazopanib is cost-effective versus sunitinib was estimated to be 95%.

The estimated cost savings from pazopanib in this analysis were based on a *post-hoc* analysis of MRU data collected in the COMPARZ trial showing lower utilisation of services associated with pazopanib compared with sunitinib treatment. Other costs during PFS were £689 lower with pazopanib than with sunitinib, largely reflecting cost savings with pazopanib versus sunitinib based on MRU data from COMPARZ. This represents a savings of approximately £49 per month of PFS and of approximately 4% of the costs of sunitinib medication. Most of these savings were associated with fewer inpatient days in the hospital. It should be noted, however, that the differences between pazopanib and sunitinib for most measures of MRU, including the number of inpatient days, were not statistically significant and that these savings are therefore associated with uncertainty. This uncertainty is reflected in the probabilistic sensitivity analyses. Also, relatively few patients in the trial were from the UK, and patterns of MRU in the COMPARZ trial may not be generalisable to patients in the UK setting.

Lacking utility data from COMPARZ, the current analysis estimated utility values by combining data on the incidence and duration of AEs in COMPARZ with a regression model that related AEs to utility values, which were estimated using data on EQ-5D utilities and AEs from the phase III pivotal trial of pazopanib. The estimated difference in mean utility values for pazopanib versus sunitinib based on this approach (0.0257) is less than that used in a prior economic evaluation from a US perspective, which was based on EQ-5D data from the PISCES trial (0.0569) [17]. While the difference in utility values for pazopanib and sunitinib in the current study is consistent with the HRQOL data from the COMPARZ trial and patient preferences in the PISCES study, the approach has limitations, and the estimated benefits of pazopanib versus sunitinib on utility values should be interpreted cautiously.

While narrow eligibility criteria improve the internal validity of clinical trials, the same criteria may decrease the generalisability of the trial results to other patients treated in other settings. In a retrospective registry study, patients with mRCC treated at academic and community practices were more likely to have poor-risk disease and impaired performance status compared with those enrolled in phase III clinical trials of new targeted therapies, including those for pazopanib and sunitinib [33]. The patient population in the current model was assumed to be consistent with that of the COMPARZ population. The characteristics of the COMPARZ patient population are not dissimilar to those of real-life treatment-naïve patients with advanced RCC seen in the clinic, suggesting that the results from this study could be largely generalisable to other populations. Additionally, the results of subgroup analyses in COMPARZ provided no evidence of the heterogeneity of treatment effects [13]. However, any generalization of the results to other populations should be made with caution.

After adjusting for the on/off-treatment schedule of sunitinib and on the basis of list prices, pazopanib and sunitinib have the same daily cost in the UK. However, pazopanib is offered at a straight 12.5% discount of the list price under the pazopanib PAS, whereas the first cycle of sunitinib is provided to the NHS for free under the sunitinib PAS. Therefore, the duration of therapy determines the effective discount for sunitinib (i.e., the longer the projected duration of therapy, the lower the effective percent discount associated with the free first cycle). Based on the duration of therapy with sunitinib estimated in the model used herein, the single free cycle of sunitinib represents a discount of roughly 14% relative to total medication costs (discounted), and the effective average medication cost per day of PFS is 0.3% lower with sunitinib than with pazopanib. If the duration of treatment with sunitinib in actual clinical practice differs from that reported in the COMPARZ trial, the effective daily cost of sunitinib and cost savings with pazopanib would be different from that reported here. The costs of sunitinib were calculated on the assumption that the NHS would receive a rebate of £3,139 for all patients who receive sunitinib. However, the mean rebate received by the NHS in reality may be lower if some providers/patients do not file the form appropriately within the permissible 60-day window, or if rebates for an entire cycle of sunitinib are not filed. The net total cost savings with pazopanib estimated in this study may therefore be conservative.

## Conclusion

The results of this study suggest that pazopanib is likely to be a cost-effective treatment option when compared with sunitinib as a first-line treatment of mRCC in the UK.
